# The Therapeutic Value of Altitude

**Published:** 1937

**Authors:** Bernard Hudson

**Affiliations:** Swiss Federal Diploma, Davos–Platz


### THE THERAPEUTIC VALUE OF ALTITUDE.

BY

BERNARD HUDSON, M.D., M.R.C.P.,
Swiss Federal Diploma,
*Davos-Platz.*

---

IT has long been a recognized fact that a holiday in the mountains exerts a remarkably invigorating and stimulating effect upon the organism, thus enabling the tired but otherwise healthy man to return to his work with renewed energy and vigour. It also puts up his resistance to disease. Since such undoubted benefits may be reaped in a short time by the normal, healthy individual, it is only natural to suppose that this climate is also likely to have a marked beneficial effect in certain cases of illness. In order to understand the "rationale" of this, it is necessary to examine briefly the physiological effects of the Alpine climate, say, at an altitude of five thousand feet, upon the organism. These physiological effects may be summarized as follows.

Owing to the height, the barometric pressure is lower than at sea-level, as a result of which the air is rarefied and the partial pressure of oxygen diminished. The effect of this is that breathing must be deeper in order that the tissues may receive an adequate amount of oxygenation. The blood, for the same reason, changes its character, and both the red cells and the hæmoglobin show an increase. This is a real augmentation, and not merely a relative increase. The metabolism of the body is maintained at a higher rate in the mountains. Other important characteristics of this climate are: the purity of the air; the complete freedom from dust, especially in the winter when the snow is on the ground; the dryness of the air; and the absence of wind. Sunlight also plays a very important part when considering the stimulating effects of the Alpine climate. Owing to the purity of the air and the absence of dust, humidity, smoke, and organisms, all of which are found in the lower strata of the air on the plains, the sunlight has much more power, and a greater bactericidal and healing effect. This effect is utilized in the treatment, by sun-bathing, of many conditions, such as tuberculosis of the bones, joints, and serous membranes and other infective conditions, for example, chronic ulcers, wounds, and fistulæ. On the other hand, sun-bathing is harmful in the majority of cases of tuberculosis of the lungs, and should never be employed except under medical control, as much damage may be done.

Acclimatization to altitude consists in the adaptation of the body to the changed meteorological conditions. The increased pulmonary ventilation, the enrichment of the blood, the response of the skin, alimentary tract and kidneys to the altered metabolic requirements establish the vital mechanism at a new level. It usually takes a week to ten days to become acclimatized, and during this period visitors, even those in good health, may suffer from slight degrees of headache, sleeplessness and shortness of breath on exertion, but these symptoms are transient and quickly pass off.

It will readily be understood from the above considerations that it is possible for the Alpine climate to exert marked beneficial and healing effects in certain cases of illness. Nevertheless, this climate may be also contra-indicated in other conditions.

The malady for which the Alpine climate has become especially famous during the last eighty or ninety years is tuberculosis of the lungs and also tuberculosis in other forms, that is, of joints, glands, bones, and serous membranes. "Direct attack" in many of these forms of tuberculous diseases is often undesirable, or impossible. "Indirect attack" then remains often the only method which we have, excepting symptomatic treatment, of combating this disease. By indirect attack is meant placing the patient in the best possible conditions of climate, rest, and nutrition, whereby his general health may be improved, thus enabling him to resist his disease, and eventually overcome it. I may here mention the undoubted beneficial effect which a sojourn in the Alps may have upon even old cases of tuberculosis with cavitation. I have seen cases with cavities in the lungs, cavities which have shown no signs of shrinking or healing for a long period in the plains, react rapidly to this climate, quickly becoming smaller, and eventually closing. I could produce many radiographs showing this undoubted fact.

The cases of tuberculosis which do the best are naturally those where the disease is not too far advanced, and the patient is still in fairly good general health with reasonable power of reaction and resistance.

In cases of surgical tuberculosis the mountain climate is eminently suitable, especially during the winter months, because sun-bathing or helio-therapy can then be carried out under the most favourable conditions. The power and radiant energy of the sun is much greater in the pure air of the mountains than in the plains, where the rays are filtered through layers of heavy, humid atmosphere, holding in suspension dust, smoke, and micro-organism. Open tuberculous lesions, such as sinuses and fistulæ in connection with bone or joint disease, often close and heal in a really remarkable manner under treatment by the mountain sun.

Other similar conditions not necessarily tuberculous, such as unclosed empyemata, sinuses, ulcers, and other septic conditions, derive much benefit from sun treatment. Cases of lupus and chronic skin diseases also may improve greatly.

With regard, however, to sun treatment a word of warning should be given. The sun scientifically employed is certainly very beneficial in cases of surgical tuberculosis and other conditions mentioned above, but it should not, as a rule, be used in pulmonary cases. There may be some types where modified sun-treatment can be employed with benefit under careful medical supervision, but as a general rule it is harmful to patients with tuberculosis of the lungs to be exposed uncovered to the sun, especially the head, neck, and chest. Congestions, hæmorrhages, and renewed activity may easily follow.

The mountain climate may be very beneficial in certain maladies of the respiratory tract other than tuberculosis. Cases of chronic bronchitis and bronchiectasis often improve in a rapid and marked manner. Bronchial asthma reacts in the most remarkable manner to the Alpine climate. The effect is often almost miraculous, and if the patient will remain for a long enough period the benefits obtained are frequently permanent. Unfortunately there is a tendency for patients when they experience great improvement, which often happens, to return home too soon. Real attacks of asthma are rarely seen in the mountains. As a result of freedom from these distressing symptoms, patients are able to sleep better. They lose that feeling of apprehension and depression, the whole outlook on life becomes more optimistic, the general health recovers, the associated bronchitis and dyspnœa abate, the heart regains its strength, and the capacity for exertion is progressively increased.

The most striking results of all are observed in children. This may be easily understood, because in such cases secondary changes such as cardiac weakness, chronic bronchitis and emphysema have not yet had time to develop, nor have actual structural alterations to the chest wall. In children especially improvement is very rapid. The attacks quickly cease, and one can see from day to day gain in health, physique and character. I can almost assert that in practically every case, if an asthmatic child is sent to live in the mountains (a height of about five thousand feet seems to be the most efficacious), the affection can certainly be permanently cured, but a stay of less than a year is not really of great permanent value, and two years are better. Education can easily be carried on, and the child does not lose time from having continually to be away from school on account of illness.

Another type of individual for whom the mountain climate is eminently suitable is the "pre-tuberculous" patient. In other words, a predisposed person in bad general health, in whom the presence of tuberculosis may even be suspected, especially if the family history is a bad one. Backward children suffering from adenoids, general debility, a tendency to colds, catarrhs and bronchitis generally do exceedingly well in a mountain climate.

Mountain climate is contra-indicated in cases where the patient is exhausted by long illness, and has very little reactive power or resistance remaining. Such cases as, for instance, those in the last stages of consumption, with fever, emaciation, asthenia, etc. The extra demand caused by the mountain climate in such cases, where there is no reactive power to meet it, only hastens the end. The occurrence of hæmorrhage and hæmoptysis is often held to be a contra-indication to sending a patient to a mountain resort. This is not generally the case. As a rule the bleeding ceases with the general improvement under treatment, and the patients do well. However, a case in the third stage, with extensive cavitation, a high blood-pressure and prone to hæmorrhage should not be sent. Cases of chronic kidney disease as a rule are not suitable for a mountain climate. Uncompensated cardiac conditions are also a contra-indication. Tuberculous ulceration of the larynx, if occurring in a person of good resisting power, as a rule does well in the mountains. It is of no use, however, sending advanced cases with extensive laryngeal involvement; the cold, dry climate only renders them more unhappy and uncomfortable, and in such cases there is no benefit.

The Alpine climate is also of great benefit in cases of convalescence after acute illness and operation. Cases of chronic malaria often receive great benefit by a residence in the mountains. There is usually a rapid recovery from the complications of this disease, complications such as debility, anæmia, œdema, albuminuria, loss of weight and cardiac weakness.

### CONCLUSIONS.

To sum up, the principal indications for treatment in the mountain climate are the following: pulmonary tuberculosis, if not too far advanced; surgical tuberculosis, that is, of bones, joints, glands and serous membranes; chronic bronchitis and bronchiectasis; convalescence after acute illness; anaemia; and, finally, the mountain climate is eminently suitable for delicate, backward children, predisposed to tuberculosis and of bad family history. Cases, for example, of poor physical development, enlarged glands, chronic bronchial trouble, asthma or adenoids. In this type of case there is nearly always a very rapid and encouraging improvement.

